# The closure property of $\mathcal{H}$-tensors under the Hadamard product

**DOI:** 10.1186/s13660-017-1499-4

**Published:** 2017-09-20

**Authors:** Lixin Zhou, Jianzhou Liu, Li Zhu

**Affiliations:** 1 School of Mathematics and Computational Science, Xiangtan University, Xiangtan, Hunan 411105 P.R. China; 2 Faculty of Science, Guilin University of Aerospace Technology, Guilin, Guangxi 541004 P.R. China; 3 Guangxi Aviation Logistics Research Center, Guilin University of Aerospace Technology, Guilin, Guangxi 541004 P.R. China

**Keywords:** 15A18, 15A69, $\mathcal{H}$-tensors, $\mathcal{M}$-tensors, Hadamard product, eigenvalues

## Abstract

In this paper, we investigate the closure property of $\mathcal {H}$-tensors under the Hadamard product. It is shown that the Hadamard products of Hadamard powers of strong $\mathcal{H}$-tensors are still strong $\mathcal{H}$-tensors. We then bound the minimal real eigenvalues of the comparison tensors of the Hadamard products involving strong $\mathcal{H}$-tensors. Finally, we show how to attain the bounds by characterizing these $\mathcal{H}$-tensors.

## Introduction

The study of tensors with their various applications has increasingly attracted extensive attention and interest [1–5]. A tensor can be regarded as a higher-order generalization of a matrix in linear algebra. However, unlike matrices, the problems for tensors are generally nonlinear. Hence, there is a large need to investigate tensor problems. Recently, some structured tensors such as nonnegative tensors, $\mathcal {M}$-tensors and $\mathcal {H}$-tensors have been introduced and studied well, and many interesting results for these tensors have been obtained because of their special structure properties [6–15]. These structural tensors have a wide range of applications such as spectral hypergraph theory, higher-order Markov chains, big amounts of data, polynomial optimization, magnetic resonance imaging, simulation, automatic control, and quantum entanglement problems [1, 2, 4–8, 10–18]. For example, the positive definiteness of an even-degree homogeneous polynomial form $f(x)$ plays an important role in the stability study of nonlinear autonomous systems via Lyapunov’s direct method in automatic control [19]. In [6], it is shown that the homogeneous polynomial form $f(x)$ is equivalent to the tensor product ${\mathcal {A}}x^{m}$ of an *m*th-order, *n*-dimensional supersymmetric tensor ${\mathcal {A}}$ and $x^{m}$, defined by the following equation (1.1) (see [4, 19]). In [16], Qi pointed out that $f(x)$ is positive definite if and only if the real supersymmetric tensor ${\mathcal {A}}$ is positive definite. For an even-order real supersymmetric tensor ${\mathcal {A}}$ of order *m* and dimension *n*, with all diagonal elements $a_{k\ldots k} > 0$, if ${\mathcal {A}}$ is an *H*-tensor, then ${\mathcal {A}}$ is positive definite [19]. The main aim of this paper is to study the closure property of structure properties of $\mathcal {H}$-tensors under the Hadamard product.

An *m*th-order *n*-dimensional real tensor $\mathcal {A}$ is a multidimensional array of $n^{m}$ real entries of the form

$$\mathcal{A}=(a_{i_{1}\ldots i_{m}}),\quad a_{i_{1}\ldots i_{m}}\in R,1\leq i_{1},\ldots, i_{m}\leq n. $$

The entries $a_{ii\ldots i}$ are called the diagonal entries of ${\mathcal {A}}$. If all its off-diagonal entries are zero, then ${\mathcal {A}}$ is diagonal. The identity tensor $\mathcal{I}$ is a diagonal tensor all of whose diagonal entries are 1. In the sequel, we denote by $\mathcal {R}^{(m,n)}$ the set of all *m*th-order *n*-dimensional real tensors. For a tensor $\mathcal {A}\in \mathcal {R}^{(m,n)}$ and a vector $x=(x_{1},\ldots ,x_{n})^{T}\in {\mathbb {C}}^{n}$, the tensor-vector multiplication ${\mathcal {A}}x^{m-1}$ is defined as an *n*-vector whose *i*th entries are

1.1 $$ \bigl({\mathcal {A}}x^{m-1}\bigr)_{i}=\sum ^{n}_{i_{2},\ldots,i_{m}=1}a_{ii_{2}\ldots i_{m}}x_{i_{2}}\ldots x_{i_{m}},\quad i=1,2,\ldots,n. $$

If there are a number *λ* and a nonzero vector $x\in {\mathbb {C}}^{n}$ such that

$${\mathcal {A}}x^{m-1}=\lambda x^{[m-1]}, $$

then *λ* is called the eigenvalue of ${\mathcal {A}}$ and *x* is the eigenvector of ${\mathcal {A}}$ associated with *λ*, where $x^{[m-1]}$ is the Hadamard power of *x*, *i.e.*, $x^{[m-1]}=(x_{1}^{m-1},\ldots ,x_{n}^{m-1})^{T}$. Note that the definition of eigenvalues of tensors was independently introduced by Qi [16] and Lim [20]. Denote by $\varphi({\mathcal {A}})$ the set of all the eigenvalues of ${\mathcal {A}}\in \mathcal {R}^{(m,n)}$, and denote

$$\rho({\mathcal {A}})=\max\bigl\{ |\lambda| | \lambda\in\varphi({\mathcal {A}})\bigr\} ,\quad\tau ({\mathcal {A}})=\min \bigl\{ \operatorname{Re}\lambda| \lambda\in\varphi({\mathcal {A}})\bigr\} , $$

where Re*λ* is the real part of *λ*. It is well known that if $\mathcal {A}\in \mathcal {R}^{(m,n)}$ is a nonnegative tensor (*i.e.*, all its entries are nonnegative), then $\rho({\mathcal {A}})$ must be its eigenvalue [13, 14]; and if $\mathcal {A}\in \mathcal {R}^{(m,n)}$ is an $\mathcal {M}$-tensor, then $\tau({\mathcal {A}})$ must be its eigenvalue [15].

A tensor $\mathcal{A}\in\mathcal{R}^{(m,n)}$ is said to be a (strong) $\mathcal {M}$-tensor if ${\mathcal {A}}$ can be written as $\mathcal {A}=s\mathcal{I}-\mathcal{B}$, where $\mathcal{B}\in\mathcal {R}^{(m,n)}$ is nonnegative and $s(>)\geq\rho(\mathcal{B})$. In this case, according to the proof of [15, Theorem 3.3], $\tau(\mathcal {A})=s-\rho(\mathcal {B})$. For a tensor $\mathcal{A}=(a_{i_{1}\ldots i_{m}})\in\mathcal {R}^{(m,n)}$, the comparison tensor $\mathcal{M}(\mathcal {A})=(m_{i_{1}\ldots i_{m}})\in \mathcal {R}^{(m,n)}$ is defined as

$$m_{i_{1}\ldots i_{m}}= \textstyle\begin{cases} |a_{i_{1}\ldots i_{m}}|,&\text{if } i_{1}=\cdots =i_{m},\\ -|a_{i_{1}\ldots i_{m}}|,&\text{otherwise}, \end{cases}\displaystyle \quad1\leq i_{1}, \ldots, i_{m}\leq n. $$

### Definition 1.1

[8, 11]

A tensor ${\mathcal {A}}\in \mathcal {R}^{(m,n)}$ is called a (strong) $\mathcal {H}$-tensor if its comparison tensor $\mathcal {M}(\mathcal{A})$ is a (strong) $\mathcal {M}$-tensor. We denote $\sigma({\mathcal {A}})=\tau(\mathcal{M}({\mathcal {A}}))$.

For a nonnegative tensor $\mathcal {A}=(a_{i_{1}i_{2}\ldots i_{m}})\in \mathcal {R}^{(m,n)}$, the matrix $R(\mathcal {A})=(r_{ij})\in {\mathbb {R}}^{n \times n}$ is called the representation of $\mathcal {A}$, where

$$r_{ij}=\sum _{\{i_{2},\ldots,i_{m}\}\ni j}a_{ii_{2}\ldots i_{m}},\quad i,j=1,2, \ldots,n. $$

### Definition 1.2

[9, 10]

A tensor $\mathcal {A}=(a_{i_{1}i_{2}\ldots i_{m}})\in \mathcal {R}^{(m,n)}$ is called weakly irreducible if the representation $R(|\mathcal {A}|)$ of $|\mathcal {A}|$ is irreducible. We denote $|\mathcal {A}|=(|a_{i_{1}i_{2}\ldots i_{m}}|)$.

Many interesting properties have been provided for $\mathcal {M}$-tensors. Recall that $\mathcal {A}\in \mathcal {R}^{(m,n)}$ is an $\mathcal {H}$-tensor if and only if $\mathcal {M}(\mathcal {A})\in \mathcal {R}^{(m,n)}$ is an $\mathcal {M}$-tensor. So using [15, Theorem 3.4] and [8, Theorem 3], we have the following facts on $\mathcal {H}$-tensors that will be frequently used in the next sections:

1. If $\mathcal {A}\in \mathcal {R}^{(m,n)}$ is an $\mathcal {H}$-tensor, then $\sigma(\mathcal {A})=\sigma(|\mathcal {A}|)$, which is the minimal real eigenvalue of $\mathcal {M}(\mathcal {A})$. Further, let $\mathcal {M}(\mathcal {A})=s\mathcal {I}-\mathcal {B}$ where $\mathcal {B}$ is nonnegative and $s\geq\rho(\mathcal {B})$. Then $\sigma(\mathcal {A})=s-\rho(\mathcal {B})$.
2. If ${\mathcal {A}}\in \mathcal {R}^{(m,n)}$ is a weakly irreducible strong $\mathcal {H}$-tensor, then $\sigma({\mathcal {A}})>0$, and there exists an *n*-vector $x>0$ such that $\mathcal{M}(\mathcal{A})x^{m-1}= \sigma({\mathcal {A}})x^{[m-1]}$.
3. A tensor ${\mathcal {A}}\in\mathcal{R}^{(m,n)}$ is a strong $\mathcal {H}$-tensor if and only if there exists an *n*-vector $x>0$ such that $\mathcal{M}(\mathcal{A})x^{m-1}>0$.

Clearly, these interesting results are due to the special structures of $\mathcal {H}$-tensors. So it is natural to consider how to preserve the structure properties under certain operations. In addition, many interesting results have been obtained for the Hadamard products involving *M*-matrices and *H*-matrices [21]. It is natural to ask whether we can provide similar results for the tensor case. Motivated by these facts, the aim of this paper is to investigate the closure property of $\mathcal {H}$-tensors under the Hadamard product.

### Definition 1.3

Given two tensors ${\mathcal {A}}=(a_{i_{1}\ldots i_{m}}),\mathcal{B}=(b_{i_{1}\ldots i_{m}})\in\mathcal{R}^{(m,n)}$, the Hadamard product of ${\mathcal {A}}$ and $\mathcal{B}$ is defined as ${\mathcal {A}}\circ\mathcal{B}=(a_{i_{1}\ldots i_{m}}b_{i_{1}\ldots i_{m}})\in \mathcal{R}^{(m,n)}$ and the *r*th Hadamard power of ${\mathcal {A}}$ is defined as ${\mathcal {A}}^{[r]}=(a^{r}_{i_{1}\ldots i_{m}})\in\mathcal{R}^{(m,n)}$ for $r\geq0$.

To obtain our results, we need the following two famous inequalities:

- *Hölder’s inequality*: let $a_{i}$ and $b_{i}$ be nonnegative numbers for $i=1,2,\ldots,n$, and let $0< r<1$. Then
  $$\sum _{i=1}^{n}a_{i}^{r}b_{i}^{1-r} \leq\Biggl(\sum _{i=1}^{n}a_{i} \Biggr)^{r}\Biggl(\sum _{i=1}^{n}b_{i} \Biggr)^{1-r}, $$
  and the equality holds if and only if, for all $i=1,2,\ldots,n$, $a_{i}=lb_{i}$ for some constant *l*.
- *Minkowski’s inequality*: let $a_{i}$ be nonnegative numbers for $i=1,2,\ldots,n$, and let $r>1$. Then
  $$\sum _{i=1}^{n}a_{i}^{r}\leq \Biggl(\sum _{i=1}^{n}a_{i} \Biggr)^{r}, $$
  and the equality holds if and only if there is at most one nonzero number for $a_{1},a_{2},\ldots,a_{n}$.

The rest of the paper is organized as follows. In Section 2, we show the closure property of the Hadamard products of Hadamard powers of strong $\mathcal {H}$-tensors. In Section 3, we bound the minimal real eigenvalues of the comparison tensors of the Hadamard products involving strong $\mathcal {H}$-tensors. In Section 4, we characterize these strong $\mathcal {H}$-tensors such that the bounds can be obtained.

## The closure property

In this section, we provide the closure property of the Hadamard products of Hadamard powers of strong $\mathcal {H}$-tensors.

### Lemma 2.1

*Let* $\mathcal {A},\mathcal {B}\in \mathcal {R}^{(m,n)}$ *be strong* $\mathcal {H}$-*tensors and let* $0\leq r\leq1$. *Then* $\mathcal {A}^{[r]}\circ \mathcal {B}^{[1-r]}$ *is a strong* $\mathcal {H}$-*tensor*.

### Proof

Set $\mathcal {A}=(a_{i_{1}i_{2}\ldots i_{m}})$ and $\mathcal {B}=(b_{i_{1}i_{2}\ldots i_{m}})$. By (P3), there exist positive vectors $x=(x_{i})\in\mathbb{R}^{n}$ and $y=(y_{i})\in\mathbb{R}^{n}$ such that $\mathcal {M}(\mathcal {A})x^{m-1}>0$ and $\mathcal {M}(\mathcal {B})y^{m-1}>0$, respectively. This means that, for all $i=1,2,\ldots,n$,

$$|a_{ii\ldots i}|x_{i}^{m-1}>\sum _{(i_{2},\ldots,i_{m})\neq (i,\ldots,i)}|a_{ii_{2}\ldots i_{m}}|x_{i_{2}} \ldots x_{i_{m}} $$

and

$$|b_{ii\ldots i}|y_{i}^{m-1}>\sum _{(i_{2},\ldots,i_{m})\neq (i,\ldots,i)}|b_{ii_{2}\ldots i_{m}}|y_{i_{2}} \ldots y_{i_{m}}. $$

Note that $0\leq r\leq1$. Thus, using the Hölder inequality, we have

$$\begin{aligned} |a_{ii\ldots i}|^{r}|b_{ii\ldots i}|^{1-r} \bigl(x_{i}^{r}y_{i}^{1-r} \bigr)^{(m-1)}>{}&\biggl(\sum _{(i_{2},\ldots ,i_{m})\neq(i,\ldots,i)}|a_{ii_{2}\ldots i_{m}}|x_{i_{2}} \ldots x_{i_{m}}\biggr)^{r} \\ &\times\biggl(\sum _{(i_{2},\ldots,i_{m})\neq (i,\ldots,i)}|b_{ii_{2}\ldots i_{m}}|y_{i_{2}}\ldots y_{i_{m}} \biggr)^{1-r} \\ \geq{}&\sum _{(i_{2},\ldots,i_{m})\neq(i,\ldots ,i)}|a_{ii_{2}\ldots i_{m}}|^{r}x^{r}_{i_{2}} \ldots x^{r}_{i_{m}}\cdot |b_{ii_{2}\ldots i_{m}}|^{1-r}y^{1-r}_{i_{2}} \ldots y^{1-r}_{i_{m}}. \end{aligned}$$

Set $z=(x^{r}_{i}y^{1-r}_{i})\in\mathbb{R}^{n}$. Then the inequality above gives $\mathcal {M}(\mathcal {A}^{[r]}\circ \mathcal {B}^{[1-r]})z^{m-1}>0$, from which it follows by (P3) that $\mathcal {A}^{[r]}\circ \mathcal {B}^{[1-r]}$ is a strong $\mathcal {H}$-tensor. The result is proved. □

### Lemma 2.2

*Let* $\mathcal {A}\in \mathcal {R}^{(m,n)}$ *be a strong* $\mathcal {H}$-*tensor and let* $t\geq1$. *Then* $\mathcal {A}^{[t]}$ *is a strong* $\mathcal {H}$-*tensor*.

### Proof

Set $\mathcal {A}=(a_{i_{1}i_{2}\ldots i_{m}})$. Clearly, there exists a positive vector $x=(x_{i})\in\mathbb{R}^{n}$ such that $\mathcal {M}(\mathcal {A})x^{m-1}>0$ and so, for all $i=1,2,\ldots,n$,

$$|a_{ii\ldots i}|x_{i}^{m-1}>\sum _{(i_{2},\ldots,i_{m})\neq (i,\ldots,i)}|a_{ii_{2}\ldots i_{m}}|x_{i_{2}} \ldots x_{i_{m}}, $$

from which we get, by considering $t\geq1$ and using the Minkowski inequality,

$$\begin{aligned} |a_{ii\ldots i}|^{t}\bigl(x_{i}^{t} \bigr)^{(m-1)}&>\biggl(\sum _{(i_{2},\ldots,i_{m})\neq(i,\ldots,i)}|a_{ii_{2}\ldots i_{m}}|x_{i_{2}} \ldots x_{i_{m}}\biggr)^{t} \\ &\geq \sum _{(i_{2},\ldots,i_{m})\neq(i,\ldots ,i)}|a_{ii_{2}\ldots i_{m}}|^{t}x^{t}_{i_{2}} \ldots x^{t}_{i_{m}}. \end{aligned}$$

Set $z=(x^{t}_{i})\in\mathbb{R}^{n}$. Then $\mathcal {M}(\mathcal {A}^{[t]})z^{m-1}>0$ and thus $\mathcal {A}^{[t]}$ is a strong $\mathcal {H}$-tensor by (P3). The result is proved. □

Now we are ready to present the main result of this section.

### Theorem 2.3

*Let* $\mathcal {A}_{1},\ldots, \mathcal {A}_{k}\in \mathcal {R}^{(m,n)}$ *be strong* $\mathcal {H}$-*tensors and let* $r_{1}, \ldots,r_{k}$ *be positive numbers with* $\sum ^{k}_{i=1}r_{i}\geq1$. *Then* $\mathcal {A}^{[r_{1}]}_{1}\circ\cdots\circ \mathcal {A}^{[r_{k}]}_{k}$ *is a strong* $\mathcal {H}$-*tensor*.

### Proof

Consider that $\mathcal {A}\in \mathcal {R}^{(m,n)}$ is a strong $\mathcal {H}$-tensor if and only if $|\mathcal {A}|\in \mathcal {R}^{(m,n)}$ is a strong $\mathcal {H}$-tensor. So, without loss of generality, assume that all the tensors $\mathcal {A}_{i}$ are nonnegative for $i=1,2,\ldots,k$. We first use the induction on *k* to prove the result in the case that $\sum ^{k}_{i=1}r_{i}=1$. Clearly, the result is true for $k=2$ by Lemma 2.1. Assume that the result is true for $k-1$. Now let

$$\mathcal {B}^{[1-r_{k}]}=\mathcal {A}^{[r_{1}]}_{1}\circ\cdots\circ \mathcal {A}^{[r_{k-1}]}_{k-1}. $$

Recall that each $\mathcal {A}_{i}$ is nonnegative. Then

$$\mathcal {B}=\mathcal {A}^{[\frac{r_{1}}{1-r_{k}}]}_{1}\circ\cdots\circ \mathcal {A}^{[\frac {r_{k-1}}{1-r_{k}}]}_{k-1}. $$

Note that $\sum _{i=1}^{k-1}\frac{r_{i}}{1-r_{k}}=1$. Hence, using the induction assumption, we conclude that $\mathcal {B}$ is a strong tensor. Further, by Lemma 2.1, $\mathcal {B}^{[1-r_{k}]}\circ \mathcal {A}^{[r_{k}]}_{k}$ is a strong $\mathcal {H}$-tensor. So the result is true in the case that $\sum ^{k}_{i=1}r_{i}=1$.

Now consider the general case $t=\sum ^{k}_{i=1}r_{i}\geq1$. Let $l_{i}=r_{i}t^{-1}$ for all $i=1,2,\ldots,k$. Then $\sum ^{k}_{i=1}l_{i}=1$. Thus, following the case above, we know that $\mathcal {C}=\mathcal {A}^{[l_{1}]}_{1}\circ\cdots\circ \mathcal {A}^{[l_{k}]}_{k}$ is a strong $\mathcal {H}$-tensor. Further, by considering $t\geq1$, using Lemma 2.2 we find that $\mathcal {C}^{[t]}=\mathcal {A}^{[r_{1}]}_{1}\circ\cdots\circ \mathcal {A}^{[r_{k}]}_{k}$ is a strong $\mathcal {H}$-tensor. The result is proved. □

### Example 2.1

Let $\mathcal {A}_{1}=(a_{ijkl}), \mathcal {A}_{2}=(b_{ijkl}), \mathcal {A}_{3}=(c_{ijkl}) \in \mathcal {R}^{(4,3)}$ be defined as follows:

$$\textstyle\begin{cases} a_{1111}=4, a_{2222}=2, a_{3333}=2, a_{1112}=a_{2111}=a_{1113}=a_{3111}=1, &\text{otherwise } a_{ijkl}=0,\\ b_{1111}=5, b_{2222}=3, b_{3333}=3, b_{1112}=b_{2111}=2, b_{1113}=b_{3111}=\frac{3}{2}, &\text{otherwise } b_{ijkl}=0,\\ c_{1111}=6, c_{2222}=3, c_{3333}=4, c_{1112}=c_{2111}=\frac{3}{2}, c_{1113}=c_{3111}=\frac{5}{2},& \text{otherwise } c_{ijkl}=0. \end{cases} $$

By (P3), it is ensured that $\mathcal {A}_{1}$, $\mathcal {A}_{2}$, and $\mathcal {A}_{3}$ are strong $\mathcal {H}$-tensors. Set $r_{1}=r_{2}=r_{3}=1$ and $x=(x_{1}, x_{2}, x_{3})^{T}=(1, 2, 2)^{T}$. Then $\mathcal {D}=\mathcal {A}^{[r_{1}]}_{1}\circ \mathcal {A}^{[r_{2}]}_{2} \circ \mathcal {A}^{[r_{3}]}_{3}=(d_{ijkl})$, where $d_{1111}=120$, $d_{2222}=18$, $d_{3333}=24$, $d_{1112}=3$, $d_{2111}=3$, $d_{1113}=\frac{15}{4}$, $d_{3111}=\frac{15}{4}$, otherwise $d_{ijkl}=0$. Since

$$\textstyle\begin{cases} |d_{1111}|x_{1}^{3}=120 \times1 =120 > |d_{1112}|x_{1}^{2}x_{2} + |d_{1113}|x_{1}^{2}x_{3}=3 \times1^{2} \times1 + \frac{15}{4} \times1^{2} \times2=\frac {21}{2},\\ |d_{2222}|x_{2}^{3}=18 \times2^{3} =144 > |d_{2111}|x_{1}^{3}=3 \times1^{3} =3,\\ |d_{3333}|x_{3}^{3}=24 \times2^{3} =192 > |d_{3111}|x_{1}^{3}=\frac{15}{4} \times1^{3} =\frac{15}{4}, \end{cases} $$

we see by (P3) that $\mathcal {D}$ is a strong $\mathcal {H}$-tensor.

## Bounding the minimal real eigenvalues

In this section, we bound the minimal real eigenvalues of the comparison tensors of the Hadamard products involving strong $\mathcal {H}$-tensors.

Let $\mathcal {A}=(a_{i_{1}i_{2}\ldots i_{m}})\in \mathcal {R}^{(m,n)}$ and let $\alpha \subseteq\{1,2,\ldots,n\}$ with $|\alpha|=k$, where $|\alpha|$ denotes the number of elements of *α*. A principal subtensor $\mathcal {A}[\alpha]$ of $\mathcal {A}$ is an *m*th-order *k*-dimensional subtensor consisting of $k^{m}$ elements defined as

$$\mathcal {A}[\alpha]=(a_{i_{1}i_{2}\ldots i_{m}}), \quad\text{where } i_{1},i_{2}, \ldots, i_{m}\in\alpha. $$

For a nonnegative tensor $\mathcal {B}\in \mathcal {R}^{(m,n)}$, let $\mathcal {B}[\alpha]$ be a principal subtensor with $|\alpha|< n$. Then $\rho(\mathcal {B}[\alpha ])\leq\rho(\mathcal {B})$ by [10, Lemma 2.2]. Further, if $\mathcal {B}$ is weakly irreducible, then $\rho(\mathcal {B}[\alpha])< \rho(\mathcal {B})$ by [12, Theorem 3.3] or [11, Proposition 2.5]. Thus we immediately have the following result.

### Lemma 3.1

*Let* $\mathcal {A}\in \mathcal {R}^{(m,n)}$ *be a strong* $\mathcal {H}$-*tensor and let* $\mathcal {A}[\alpha]$ *be a principal subtensor with* $|\alpha|< n$. *Then* $\mathcal {A}[\alpha]$ *is a strong* $\mathcal {H}$-*tensor and* $\sigma(\mathcal {A}[\alpha])\geq\sigma(\mathcal {A})$. *Furthermore*, *if* $\mathcal {A}$ *is weakly irreducible*, *then* $\sigma(\mathcal {A}[\alpha])> \sigma(\mathcal {A})$.

### Proof

Let $\mathcal {M}(\mathcal {A})=s\mathcal {I}-\mathcal {B}$, where $\mathcal {B}$ is a nonnegative tensor and $s>\rho(\mathcal {B})$. Then $\mathcal {M}(\mathcal {A}[\alpha])=s\mathcal {I}-\mathcal {B}[\alpha ]$ and $s-\rho(\mathcal {B}[\alpha])\geq s-\rho(\mathcal {B})>0$. So $\mathcal {A}[\alpha]$ is a strong $\mathcal {H}$-tensor with $\sigma(\mathcal {A}[\alpha])\geq\sigma(\mathcal {A})$. Further, if $\mathcal {A}$ is weakly irreducible, then $\mathcal {B}$ is also weakly irreducible by Definition 1.2, so $\rho(\mathcal {B}[\alpha ])<\rho(\mathcal {B})$, which implies that $\sigma(\mathcal {A}[\alpha])> \sigma (\mathcal {A})$. The result is proved. □

For a nonnegative tensor $\mathcal {B}\in \mathcal {R}^{(m,n)}$, by [10, Theorem 5.2], there exists a partition $\{\alpha_{1},\ldots,\alpha_{p}\} $ of $\{1,2,\ldots,n\}$ such that the principal subtensor $\mathcal {B}[\alpha _{i}]$ is weakly irreducible for $i=1,2,\ldots,p$. Also, $\rho(\mathcal {B})=\rho(\mathcal {B}[\alpha_{t}])$ for some $1\leq t\leq p$. Thus we immediately have the following result.

### Lemma 3.2

*Let* $\mathcal {A}\in \mathcal {R}^{(m,n)}$ *be a strong* $\mathcal {H}$-*tensor*. *Then there exists* $\alpha\subseteq\{1,2,\ldots,n\}$ *such that* $\mathcal {A}[\alpha]$ *is a weakly irreducible strong* $\mathcal {H}$-*tensor with* $\sigma(\mathcal {A})=\sigma(\mathcal {A}[\alpha])$.

### Proof

Let $\mathcal {M}(\mathcal {A})=s\mathcal {I}-\mathcal {B}$, where $\mathcal {B}$ is a nonnegative tensor and $s>\rho(\mathcal {B})$. Assume that $\mathcal {B}[\alpha]$ is a weakly irreducible principal subtensor of $\mathcal {B}$ such that $\rho(\mathcal {B})=\rho (\mathcal {B}[\alpha])$. Then, by Definition 1.2 and Lemma 3.1, $\mathcal {A}[\alpha]$ is a weakly irreducible strong $\mathcal {H}$-tensor. Moreover, $\sigma(\mathcal {A})=s-\rho(\mathcal {B})=s-\rho(\mathcal {B}[\alpha])=\sigma (\mathcal {A}[\alpha])$. The result is proved. □

### Lemma 3.3

[13, Lemma 5.3]

*Let* $\mathcal {B}\in \mathcal {R}^{(m,n)}$ *be a nonnegative tensor and let* $x=(x_{i})\in\mathbb {R}^{n}$ *be a positive vector*. *Then*

$$\min _{1\leq i\leq n}\frac{(\mathcal {B}x^{m-1})_{i}}{x_{i}^{m-1}}\leq \rho(\mathcal {B})\leq\max _{1\leq i\leq n} \frac{(\mathcal {B}x^{m-1})_{i}}{x_{i}^{m-1}}. $$

### Lemma 3.4

*Let* $\mathcal {A}\in \mathcal {R}^{(m,n)}$ *be an* $\mathcal {M}$-*tensor and let* $\mathcal {A}z^{m-1}\geq k z^{[m-1]}$ *for a positive vector* $z\in\mathbb{R}^{n}$. *Then* $\tau(\mathcal {A})\geq k$.

### Proof

Let $\mathcal {A}=s\mathcal {I}-\mathcal {B}$, where $\mathcal {B}$ is a nonnegative tensor and $s\geq\rho(\mathcal {B})$. Since $\mathcal {A}z^{m-1}\geq k z^{[m-1]}$ for $z=(z_{i})\in\mathbb{R}^{n}>0$, we have, for all $i=1,2,\ldots,n$,

$$sz_{i}^{m-1}-\bigl(\mathcal {B}z^{m-1}\bigr)_{i} \geq k z_{i}^{m-1}, $$

from which it follows that

$$\max _{1\leq i\leq n}\frac{(\mathcal {B}z^{m-1})_{i}}{z_{i}^{m-1}}\leq s-k. $$

So, by Lemma 3.3, $\rho(\mathcal {B})\leq s-k$. Thus $\tau(\mathcal {A})=s-\rho(\mathcal {B})\geq k$. The result is proved. □

### Lemma 3.5

*Let* $\mathcal {A},\mathcal {B}\in \mathcal {R}^{(m,n)}$ *be strong* $\mathcal {H}$-*tensors*, *and let* $0\leq r\leq1$. *Then*

3.1 $$ \sigma\bigl(\mathcal {A}^{[r]}\circ \mathcal {B}^{[1-r]}\bigr)\geq \sigma(\mathcal {A})^{r}\sigma(\mathcal {B})^{1-r}. $$

### Proof

The result is trivial for $r=0,1$. So let $0< r<1$. We first consider the case where $\mathcal {A}^{[r]}\circ \mathcal {B}^{[1-r]}$ is weakly irreducible. Obviously, both $\mathcal {A}$ and $\mathcal {B}$ must be weakly irreducible. Thus, by (P2), there exist positive eigenvectors $x=(x_{i})\in\mathbb{R}^{n}$ and $y=(y_{i})\in\mathbb{R}^{n}$ such that $\mathcal {M}(\mathcal {A})x^{m-1}=\sigma(\mathcal {A})x^{[m-1]}$ and $\mathcal {M}(\mathcal {B})y^{m-1}=\sigma(\mathcal {B})y^{[m-1]}$, respectively. Let $\mathcal {A}=(a_{i_{1}i_{2}\ldots i_{m}})$ and $\mathcal {B}=(b_{i_{1}i_{2}\ldots i_{m}})$. Then, for all $i=1,2,\ldots,n$,

3.2 $$ \textstyle\begin{cases}|a_{ii\ldots i}|x_{i}^{m-1}-\sum _{(i_{2},\ldots ,i_{m})\neq(i,\ldots,i)}|a_{ii_{2}\ldots i_{m}}|x_{i_{2}}\ldots x_{i_{m}}=\sigma(\mathcal {A})x_{i}^{m-1}>0,\\ |b_{ii\ldots i}|y_{i}^{m-1}-\sum _{(i_{2},\ldots,i_{m})\neq (i,\ldots,i)}|b_{ii_{2}\ldots i_{m}}|y_{i_{2}}\ldots y_{i_{m}}=\sigma (\mathcal {B})y_{i}^{m-1}>0. \end{cases} $$

Set $z=(x^{r}_{i}y^{1-r}_{i})\in\mathbb{R}^{n}$. Then, by the Hölder inequality, we have, for all $i=1,2,\ldots,n$,

3.3 $$\begin{aligned}[b] \bigl(\mathcal {M}\bigl(\mathcal {A}^{[r]}\circ \mathcal {B}^{[1-r]}\bigr)z^{m-1} \bigr)_{i} ={} & \bigl(|a_{ii\ldots i}|x_{i}^{m-1} \bigr)^{r}\bigl(|b_{ii\ldots i}|y_{i}^{m-1} \bigr)^{1-r} \\ & -\sum _{(i_{2},\ldots,i_{m})\neq(i,\ldots ,i)}\bigl(|a_{ii_{2}\ldots i_{m}}|x_{i_{2}}\ldots x_{i_{m}}\bigr)^{r}\bigl(|b_{ii_{2}\ldots i_{m}}|y_{i_{2}}\ldots y_{i_{m}}\bigr)^{1-r} \\ \geq{}& \bigl(|a_{ii\ldots i}|x_{i}^{m-1} \bigr)^{r}\bigl(|b_{ii\ldots i}|y_{i}^{m-1} \bigr)^{1-r} \\ & -\biggl(\sum _{(i_{2},\ldots,i_{m})\neq(i,\ldots ,i)}|a_{ii_{2}\ldots i_{m}}|x_{i_{2}} \ldots x_{i_{m}}\biggr)^{r}\\ &\times\biggl(\sum _{(i_{2},\ldots,i_{m})\neq(i,\ldots,i)}|b_{ii_{2}\ldots i_{m}}|y_{i_{2}}\ldots y_{i_{m}} \biggr)^{1-r} \\ \geq{}& \biggl(|a_{ii\ldots i}|x_{i}^{m-1}- \sum _{(i_{2},\ldots ,i_{m})\neq(i,\ldots,i)}|a_{ii_{2}\ldots i_{m}}|x_{i_{2}}\ldots x_{i_{m}}\biggr)^{r} \\ &\times \biggl(|b_{ii\ldots i}|y_{i}^{m-1}- \sum _{(i_{2},\ldots ,i_{m})\neq(i,\ldots,i)}|b_{ii_{2}\ldots i_{m}}|y_{i_{2}}\ldots y_{i_{m}}\biggr)^{1-r} \\ ={}&\bigl(\sigma(\mathcal {A})x_{i}^{m-1} \bigr)^{r}\bigl(\sigma(\mathcal {B})y_{i}^{m-1} \bigr)^{1-r}=\sigma(\mathcal {A})^{r}\sigma(\mathcal {B})^{1-r}z_{i}^{m-1}. \end{aligned} $$

So $\mathcal {M}(\mathcal {A}^{[r]}\circ \mathcal {B}^{[1-r]})z^{m-1}\geq\sigma(\mathcal {A})^{r}\sigma(\mathcal {B})^{1-r} z^{[m-1]}$ for $z>0$. Consider that $\mathcal {A}^{[r]}\circ \mathcal {B}^{[1-r]}$ is a strong $\mathcal {H}$-tensor by Theorem 2.3. Thus, using Lemma 3.4, we get $\sigma(\mathcal {A}^{[r]}\circ \mathcal {B}^{[1-r]})\geq\sigma(\mathcal {A})^{r}\sigma(\mathcal {B})^{1-r}$.

Now we consider the general case. Recall that $\mathcal {A}^{[r]}\circ \mathcal {B}^{[1-r]}$ is a strong $\mathcal {H}$-tensor. By Lemma 3.2, there exists $\alpha\subseteq\{1,2,\ldots,n\}$ such that $(\mathcal {A}^{[r]}\circ \mathcal {B}^{[1-r]})[\alpha]=(\mathcal {A}[\alpha])^{[r]}\circ(\mathcal {B}[\alpha])^{[1-r]}$ is a weakly irreducible $\mathcal {H}$-tensor with $\sigma(\mathcal {A}^{[r]}\circ \mathcal {B}^{[1-r]})=\sigma((\mathcal {A}^{[r]}\circ \mathcal {B}^{[1-r]})[\alpha])$. Note that $\mathcal {A}[\alpha]$ and $\mathcal {B}[\alpha]$ are strong $\mathcal {H}$-tensors. Thus, according to the case above, using Lemma 3.1 we get

$$\sigma\bigl(\mathcal {A}^{[r]}\circ \mathcal {B}^{[1-r]}\bigr)=\sigma\bigl(\bigl( \mathcal {A}[\alpha ]\bigr)^{[r]}\circ\bigl(\mathcal {B}[\alpha]\bigr)^{[1-r]}\bigr) \geq\sigma\bigl(\mathcal {A}[\alpha ]\bigr)^{r}\sigma\bigl(\mathcal {B}[\alpha] \bigr)^{1-r}\geq\sigma(\mathcal {A})^{r}\sigma(\mathcal {B})^{1-r}. $$

The result is proved. □

### Lemma 3.6

*Let* $\mathcal {A}\in \mathcal {R}^{(m,n)}$ *be a strong* $\mathcal {H}$-*tensor*, *and let* $t\geq1$. *Then* $\sigma(\mathcal {A}^{[t]})\geq\sigma (\mathcal {A})^{t}$.

### Proof

First assume that $\mathcal {A}^{[t]}$ is weakly irreducible. Obviously, $\mathcal {A}$ is weakly irreducible. Then by (P2), there exists a positive eigenvector $x=(x_{i})\in\mathbb{R}^{n}$ such that $\mathcal {M}(\mathcal {A})x^{m-1}=\sigma(\mathcal {A})x^{[m-1]}$. Let $\mathcal {A}=(a_{i_{1}i_{2}\ldots i_{m}})$. Then, for all $i=1,2,\ldots,n$,

3.4 $$ |a_{ii\ldots i}|x^{m-1}_{i}-\sum _{(i_{2},\ldots,i_{m})\neq(i,\ldots,i)}|a_{ii_{2}\ldots i_{m}}|x_{i_{2}}\ldots x_{i_{m}}=\sigma( \mathcal {A})x^{m-1}_{i}>0. $$

Set $z=(x^{t}_{i})\in {\mathbb {R}}^{n}$. Then, by the Minkowski inequality, we have, for all $i=1,2,\ldots,n$,

3.5 $$\begin{aligned}[b] \bigl(\mathcal {M}\bigl(\mathcal {A}^{[t]}\bigr)z^{m-1}\bigr)_{i}&=\big|a^{t}_{ii\ldots i}\big| \bigl(x^{t}_{i}\bigr)^{m-1}-\sum _{(i_{2},\ldots,i_{m})\neq(i,\ldots ,i)}\big|a^{t}_{ii_{2}\ldots i_{m}}\big|x^{t}_{i_{2}} \ldots x^{t}_{i_{m}} \\ &\geq\bigl( |a_{ii\ldots i}|x_{i}^{m-1} \bigr)^{t}-\biggl(\sum _{(i_{2},\ldots ,i_{m})\neq(i,\ldots,i)}|a_{ii_{2}\ldots i_{m}}|x_{i_{2}} \ldots x_{i_{m}}\biggr)^{t} \\ &\geq \biggl(|a_{ii\ldots i}|x_{i}^{m-1}- \sum _{(i_{2},\ldots ,i_{m})\neq(i,\ldots,i)}|a_{ii_{2}\ldots i_{m}}|x_{i_{2}}\ldots x_{i_{m}}\biggr)^{t} \\ &=\sigma(\mathcal {A})^{t}z^{m-1}_{i}.\end{aligned} $$

So $\mathcal {M}(\mathcal {A}^{[t]})z^{m-1}\geq\sigma(\mathcal {A})^{t}z^{[m-1]}$ for $z>0$. Consider that $\mathcal {A}^{[t]}$ is a strong $\mathcal {H}$-tensor by Lemma 2.2. Thus, using Lemma 3.4, we get $\sigma(\mathcal {A}^{[t]})\geq \sigma(\mathcal {A})^{t}$.

Now we consider the general case. Recall that $\mathcal {A}^{[t]}$ is a strong $\mathcal {H}$-tensor. By Lemma 3.2, there exists $\alpha\subseteq\{ 1,2,\ldots,n\}$ such that $\mathcal {A}^{[t]}[\alpha]=(\mathcal {A}[\alpha])^{[t]}$ is a weakly irreducible $\mathcal {H}$-tensor with $\sigma(\mathcal {A}^{[t]})=\sigma (\mathcal {A}^{[t]}[\alpha])$. Thus, according to the case above, using Lemma 3.1 we get

$$\sigma\bigl(\mathcal {A}^{[t]}\bigr)=\sigma\bigl(\bigl(\mathcal {A}[\alpha] \bigr)^{[t]}\bigr)\geq\sigma\bigl(\mathcal {A}[\alpha]\bigr)^{t}\geq \sigma(\mathcal {A})^{t}. $$

The result is proved. □

Our main result of this section is the following.

### Theorem 3.7

*Let* $\mathcal {A}_{1},\mathcal {A}_{2},\ldots, \mathcal {A}_{k}\in \mathcal {R}^{(m,n)}$ *be strong* $\mathcal {H}$-*tensors and let* $r_{1},r_{2},\ldots,r_{k}$ *be positive numbers such that* $\sum ^{k}_{i=1}r_{i}\geq1$. *Then*

3.6 $$ \sigma\bigl(\mathcal {A}_{1}^{[r_{1}]}\circ \mathcal {A}_{2}^{[r_{2}]}\circ\cdots\circ \mathcal {A}_{k}^{[r_{k}]} \bigr)\geq\sigma(\mathcal {A}_{1})^{r_{1}}\sigma(\mathcal {A}_{2})^{r_{2}} \cdots\sigma(\mathcal {A}_{k})^{r_{k}}. $$

### Proof

By (P1), without loss of generality, assume that all the tensors $\mathcal {A}_{i}$ are nonnegative for $i=1,2,\ldots,k$. We first use the induction on *k* to prove the result in the case that $\sum ^{k}_{i=1}r_{i}=1$. Obviously, the result is true for $k=2$ by Lemma 3.5. Assume the result is true for $k-1$. Now let

$$\mathcal {B}^{[1-r_{k}]}=\mathcal {A}_{1}^{[r_{1}]}\circ\cdots\circ \mathcal {A}_{k-1}^{[r_{k-1}]}. $$

Consider that each $\mathcal {A}_{i}$ is nonnegative. Then

$$\mathcal {B}=\mathcal {A}_{1}^{[\frac{r_{1}}{1-r_{k}}]}\circ\cdots\circ \mathcal {A}_{k-1}^{[\frac{r_{k-1}}{1-r_{k}}]}. $$

Note that $\sum _{i=1}^{k-1}\frac{r_{i}}{1-r_{k}}=1$. Thus $\mathcal {B}$ is a strong $\mathcal {H}$-tensor by Theorem 2.3. Therefore, using the induction assumption, we get

3.7 $$\begin{aligned}[b] \sigma\bigl(\mathcal {A}_{1}^{[r_{1}]}\circ \mathcal {A}_{2}^{[r_{2}]} \circ \cdots\circ \mathcal {A}_{k}^{[r_{k}]}\bigr)&=\sigma\bigl( \mathcal {B}^{[1-r_{k}]}\circ \mathcal {A}_{k}^{[r_{k}]}\bigr)\geq\sigma( \mathcal {B})^{1-r_{k}}\sigma( \mathcal {A}_{k})^{r_{k}} \\ &\geq\bigl( \sigma(\mathcal {A}_{1})^{\frac{r_{1}}{1-r_{k}}}\cdots\sigma(\mathcal {A}_{k-1})^{\frac{r_{k-1}}{1-r_{k}}} \bigr)^{1-r_{k}}\sigma( \mathcal {A}_{k})^{r_{k}} \\ &=\sigma( \mathcal {A}_{1})^{r_{1}}\cdots\sigma(\mathcal {A}_{k-1})^{r_{k-1}} \sigma (\mathcal {A}_{k})^{r_{k}}. \end{aligned} $$

So the result is true in the case that $\sum ^{k}_{i=1}r_{i}=1$.

Now we consider the general case $t=\sum ^{k}_{i=1}r_{i}\geq 1$. Set $l_{i}=r_{i}t^{-1}$ for $i=1,2,\ldots,k$. Then $\sum ^{k}_{i=1}l_{i}=1$. Thus $\mathcal {C}=\mathcal {A}_{1}^{[l_{1}]}\circ \mathcal {A}_{2}^{[l_{2}]}\circ\cdots\circ \mathcal {A}_{k}^{[l_{k}]}$ is a strong $\mathcal {H}$-tensor by Theorem 2.3. Therefore, according to the case above, using Lemma 3.6 we get

$$\begin{aligned} \sigma\bigl(\mathcal {A}_{1}^{[r_{1}]}\circ \mathcal {A}_{2}^{[r_{2}]} \circ\cdots\circ \mathcal {A}_{k}^{[r_{k}]}\bigr)&=\sigma\bigl( \mathcal {C}^{[t]}\bigr) \geq\sigma(\mathcal {C})^{t} \\ &\geq \bigl(\sigma(\mathcal {A}_{1})^{l_{1}}\sigma( \mathcal {A}_{2})^{l_{2}}\cdots \sigma(\mathcal {A}_{k})^{l_{k}} \bigr)^{t} \\ &=\sigma(\mathcal {A}_{1})^{r_{1}}\sigma(\mathcal {A}_{2})^{r_{2}} \cdots\sigma(\mathcal {A}_{k})^{r_{k}}.\end{aligned} $$

The result is proved. □

### Example 3.1

Let $\mathcal {A}_{1}=(a_{ijkl}), \mathcal {A}_{2}=(b_{ijkl}), \mathcal {A}_{3}=(c_{ijkl}) \in \mathcal {R}^{(4,2)}$ be defined as follows:

$$\textstyle\begin{cases} a_{1111}=4, a_{1112}=a_{2111}=a_{1211}=a_{1121}=1, a_{2222}=2, &\text{otherwise } a_{ijkl}=0,\\ b_{1111}=5, b_{1112}=b_{2111}=b_{1211}=b_{1121}=1, b_{2222}=4, &\text{otherwise } b_{ijkl}=0,\\ c_{1111}=6, c_{1112}=a_{2111}=c_{1211}=c_{1121}=1, c_{2222}=4, & \text{otherwise } c_{ijkl}=0. \end{cases} $$

By (P3), it is assured that $\mathcal {A}_{1}$, $\mathcal {A}_{2}$, and $\mathcal {A}_{3}$ are strong $\mathcal {H}$-tensors. Now set $r_{1}=r_{2}=r_{3}=1$. Then $\mathcal {D}=\mathcal {A}^{[r_{1}]}_{1}\circ \mathcal {A}^{[r_{2}]}_{2} \circ \mathcal {A}^{[r_{3}]}_{3}=(d_{ijkl})$, where $d_{1111}=120$, $d_{2222}=32$, $d_{1112}=1$, $d_{2111}=1$, $d_{1211}=1$, $d_{1121}=1$, otherwise $d_{ijkl}=0$. By Corollary 2 of Qi [16], we get

$$\textstyle\begin{cases} \varphi[M({\mathcal {A}}_{1})]=\{ 1, 2, 2, 3.547+2.125i, 3.547-2.125i, 5.905 \},\\ \varphi[M(\mathcal {A}_{2})]=\{ 2.422, 4, 4, 4.756+2.239i, 4.756-2.239i, 7.065 \},\\ \varphi[M(\mathcal {A}_{3})]=\{ 3, 4, 4, 5.547+2.125i, 5.547-2.125i, 7.905 \},\\ \varphi[M(\mathcal {D})]=\{ 31.999, 32, 32, 119.663+0.585i, 119.663-0.585i, 120.672 \}. \end{cases} $$

So $\sigma(\mathcal {D})=31.999\geq\sigma(\mathcal {A}_{1})\sigma(\mathcal {A}_{2}) \sigma (\mathcal {A}_{3})=1 \times2.422 \times3=7.266$.

## Characterizations for the equality case

In this section, we characterize the strong $\mathcal {H}$-tensors such that the equality of (3.6) holds.

### Lemma 4.1

[12, Lemma 3.2]

*Let* $\mathcal {B}\in \mathcal {R}^{(m,n)}$ *be a weakly irreducible nonnegative tensor and let* $\mathcal {B}z^{m-1}\leq\rho(\mathcal {B}) z^{[m-1]}$ *for a positive vector* $z\in\mathbb {R}^{n}$. *Then* $\mathcal {B}z^{m-1}=\rho(\mathcal {B}) z^{[m-1]}$.

Using Lemma 4.1, we immediately get the following result.

### Lemma 4.2

*Let* $\mathcal {A}\in \mathcal {R}^{(m,n)}$ *be a weakly irreducible strong* $\mathcal {M}$-*tensor and let* $\mathcal {A}z^{m-1}\geq\tau(\mathcal {A}) z^{[m-1]}$ *for a positive vector* $z\in\mathbb{R}^{n}$. *Then* $\mathcal {A}z^{m-1}=\tau(\mathcal {A}) z^{[m-1]}$.

### Proof

Let $\mathcal {A}=s\mathcal {I}-\mathcal {B}$, where $\mathcal {B}$ is a nonnegative tensor and $s> \rho(\mathcal {B})$. Obviously, $\mathcal {B}$ is weakly irreducible. Since $\mathcal {A}z^{m-1}\geq\tau(\mathcal {A}) z^{[m-1]}$ where $\tau(\mathcal {A})=s-\rho(\mathcal {B})$, we have $\mathcal {B}z^{m-1}\leq\rho(\mathcal {B}) z^{[m-1]}$ for $z>0$. Thus, by Lemma 4.1, $\mathcal {B}z^{m-1}= \rho(\mathcal {B}) z^{[m-1]}$. So $\mathcal {A}z^{m-1}=\tau(\mathcal {A}) z^{[m-1]}$. The result is proved. □

For a tensor $\mathcal {A}=(a_{i_{1}i_{2}\ldots i_{m}})\in \mathcal {R}^{(m,n)}$ and a nonsingular diagonal matrix $D=\operatorname{diag}(d_{ii})\in {\mathbb {R}}^{n \times n}$, the tensor $\mathcal {C}= \mathcal {A}D^{-(m-1)}\cdot\underbrace{D\cdots D}_{m-1}=(c_{i_{1}i_{2}\ldots i_{m}})\in \mathcal {R}^{(m,n)}$ is defined as

$$c_{i_{1}i_{2}\ldots i_{m}}=a_{i_{1}i_{2}\ldots i_{m}}d^{-(m-1)}_{i_{1},i_{1}}d_{i_{2},i_{2}} \cdots d_{i_{m},i_{m}}, \quad1\leq i_{1},i_{2},\ldots, i_{m}\leq n. $$

It must be pointed out that $\mathcal {A}$ and $\mathcal {C}$ have the same eigenvalues [13]. In particular, if $\mathcal {A}$ and $\mathcal {C}$ are strong $\mathcal {H}$-tensors, then $\mathcal {M}(\mathcal {C})= \mathcal {M}(\mathcal {A}) |D|^{-(m-1)}\cdot\underbrace {|D|\cdots|D|}_{m-1}$, so $\sigma(\mathcal {A})=\sigma(\mathcal {C})$.

### Lemma 4.3

*Let* $\mathcal {A},\mathcal {B}\in \mathcal {R}^{(m,n)}$ *be weakly irreducible strong* $\mathcal {H}$-*tensors and let* $0< r< 1$. *Then*

$$\sigma\bigl(\mathcal {A}^{[r]}\circ \mathcal {B}^{[1-r]}\bigr)= \sigma( \mathcal {A})^{r}\sigma(\mathcal {B})^{1-r} $$

*if and only if there exist* $\gamma>0$ *and a positive diagonal matrix* $D\in {\mathbb {R}}^{n \times n}$ *such that*

$$|\mathcal {A}|=\gamma|\mathcal {B}| D^{-(m-1)}\cdot\underbrace{D\cdots D}_{m-1}. $$

### Proof

As regards sufficiency, we have $\sigma(\mathcal {A})^{r}\sigma(\mathcal {B})^{1-r}=\gamma^{r} \sigma(\mathcal {B})^{r}\sigma(\mathcal {B})^{1-r}=\gamma^{r} \sigma(\mathcal {B})$ and

$$\begin{aligned} \sigma\bigl(\mathcal {A}^{[r]}\circ \mathcal {B}^{[1-r]}\bigr)&=\sigma\bigl(|\mathcal {A}|^{[r]}\circ|\mathcal {B}|^{[1-r]}\bigr)\\ &=\sigma\bigl(\gamma^{r} \bigl(|\mathcal {B}|^{[r]}\circ|\mathcal {B}|^{[1-r]}\bigr) \bigl(D^{r} \bigr)^{-(m-1)}\cdot\underbrace{D^{r}\cdots D^{r}}_{m-1} \bigr)=\gamma^{r} \sigma(\mathcal {B}),\end{aligned} $$

and thus the sufficiency is true.

Necessarily, according to the proof of Lemma 3.5, there exists $\alpha\subseteq\{1,2,\ldots,n\}$ such that $(\mathcal {A}^{[r]}\circ \mathcal {B}^{[1-r]})[\alpha]$ is a weakly irreducible $\mathcal {H}$-tensor and

$$\begin{aligned} \sigma\bigl(\mathcal {A}^{[r]}\circ \mathcal {B}^{[1-r]}\bigr)&=\sigma\bigl(\bigl( \mathcal {A}^{[r]}\circ \mathcal {B}^{[1-r]}\bigr)[\alpha]\bigr)\\&=\sigma\bigl(\bigl( \mathcal {A}[\alpha]\bigr)^{[r]}\circ\bigl(\mathcal {B}[\alpha ]\bigr)^{[1-r]}\bigr) \geq\sigma\bigl(\mathcal {A}[\alpha]\bigr)^{r}\sigma\bigl(\mathcal {B}[\alpha] \bigr)^{1-r}. \end{aligned}$$

Recall that $\mathcal {A}=(a_{i_{1}i_{2}\ldots i_{m}})$ and $\mathcal {B}=(b_{i_{1}i_{2}\ldots i_{m}})$ are weakly irreducible strong $\mathcal {H}$-tensors. Thus, if $|\alpha|< n$, then, by Lemma 3.1, $\sigma(\mathcal {A}[\alpha ])>\sigma(\mathcal {A})$ and $\sigma(\mathcal {B}[\alpha])>\sigma(\mathcal {B})$, from which it follows that $\sigma(\mathcal {A}^{[r]}\circ \mathcal {B}^{[1-r]})>\sigma (\mathcal {A})^{r}\sigma(\mathcal {B})^{1-r}$, a contradiction. So $|\alpha|=n$. Hence, $\mathcal {A}^{[r]}\circ \mathcal {B}^{[1-r]}$ must be weakly irreducible and thus, according to the proof of Lemma 3.5, (3.3) is true, *i.e.*,

$$\begin{aligned} \mathcal {M}\bigl(\mathcal {A}^{[r]}\circ \mathcal {B}^{[1-r]}\bigr)z^{m-1}&\geq \sigma(\mathcal {A})^{r}\sigma (\mathcal {B})^{1-r} z^{[m-1]}\\&=\sigma\bigl( \mathcal {A}^{[r]}\circ \mathcal {B}^{[1-r]}\bigr)z^{[m-1]}, \quad 0< z= \bigl(x^{r}_{i}y^{1-r}_{i}\bigr)\in \mathbb{R}^{n}, \end{aligned}$$

from which it follows by Lemma 4.2 that

$$\mathcal {M}\bigl(\mathcal {A}^{[r]}\circ \mathcal {B}^{[1-r]}\bigr)z^{m-1}=\sigma( \mathcal {A})^{r}\sigma(\mathcal {B})^{1-r} z^{[m-1]}. $$

This means that the two Hölder inequalities of (3.3) are equalities and so, for all $i=1,2,\ldots,n$,

$$|a_{ii_{2}\ldots i_{m}}|x_{i_{2}}\ldots x_{i_{m}}=k_{i}|b_{ii_{2}\ldots i_{m}}|y_{i_{2}} \ldots y_{i_{m}},\quad\forall (i_{2},\ldots ,i_{m}) \neq(i,\ldots,i) $$

for some constant $k_{i}$ and for some constant $l_{i}$

$$\textstyle\begin{cases}\sum _{(i_{2},\ldots,i_{m})\neq(i,\ldots ,i)}|a_{ii_{2}\ldots i_{m}}|x_{i_{2}}\ldots x_{i_{m}}= l_{i}\sum _{(i_{2},\ldots,i_{m})\neq(i,\ldots,i)}|b_{ii_{2}\ldots i_{m}}|y_{i_{2}}\ldots y_{i_{m}},\\ |a_{ii\ldots i}|x_{i}^{m-1}-\sum _{(i_{2},\ldots,i_{m})\neq (i,\ldots,i)}|a_{ii_{2}\ldots i_{m}}|x_{i_{2}}\ldots x_{i_{m}}\\ \quad=l_{i}(|b_{ii\ldots i}|y_{i}^{m-1}-\sum _{(i_{2},\ldots,i_{m})\neq(i,\ldots,i)}|b_{ii_{2}\ldots i_{m}}|y_{i_{2}}\ldots y_{i_{m}}), \end{cases} $$

from which we get $k_{i}=l_{i}$ and

$$|a_{ii_{2}\ldots i_{m}}|x_{i_{2}}\ldots x_{i_{m}}=k_{i}|b_{ii_{2}\ldots i_{m}}|y_{i_{2}} \ldots y_{i_{m}},\quad\forall i, i_{2},\ldots,i_{m}. $$

By considering (3.2),

$$\sigma(\mathcal {A})x_{i}^{m-1}=k_{i}\sigma( \mathcal {B})y_{i}^{m-1}\quad\Rightarrow \quad k_{i}= \frac{\sigma(\mathcal {A})}{\sigma(\mathcal {B})}\frac {x_{i}^{m-1}}{y_{i}^{m-1}}. $$

Therefore we have, for all $i=1,2,\ldots,n$,

4.1 $$ |a_{ii_{2}\ldots i_{m}}|=|b_{ii_{2}\ldots i_{m}}|\frac{\sigma(\mathcal {A})}{\sigma(\mathcal {B})} \frac {x_{i}^{m-1}}{y_{i}^{m-1}}\frac{y_{i_{2}}}{x_{i_{2}}}\cdots\frac {y_{i_{m}}}{x_{i_{m}}},\quad1\leq i_{2},\ldots,i_{m}\leq n. $$

Set $D=\operatorname{diag}(\frac{y_{1}}{x_{1}},\ldots,\frac{y_{n}}{x_{n}})\in {\mathbb {R}}^{n \times n}$ and $\gamma=\frac{\sigma(\mathcal {A})}{\sigma(\mathcal {B})}$. Then (4.1) implies that $|\mathcal {A}|=\gamma|\mathcal {B}| D^{-(m-1)}\cdot \underbrace{D\cdots D}_{m-1}$. The result is proved. □

Now we characterize strong $\mathcal {H}$-tensors such that the equality of (3.6) holds in the case that $\sum ^{k}_{i=1}r_{i}=1$.

### Theorem 4.4

*Let* $\mathcal {A}_{1},\mathcal {A}_{2},\ldots, \mathcal {A}_{k}\in \mathcal {R}^{(m,n)}$ *be strong* $\mathcal {H}$-*tensors and let* $r_{1},r_{2},\ldots,r_{k}$ *be positive numbers such that* $\sum ^{k}_{i=1}r_{i}= 1$. *Then*

$$\sigma\bigl(\mathcal {A}_{1}^{[r_{1}]}\circ \mathcal {A}_{2}^{[r_{2}]} \circ\cdots\circ \mathcal {A}_{k}^{[r_{k}]}\bigr)=\sigma( \mathcal {A}_{1})^{r_{1}}\sigma(\mathcal {A}_{2})^{r_{2}}\cdots \sigma(\mathcal {A}_{k})^{r_{k}} $$

*if and only if there exists* $\alpha\subseteq\{1,2,\ldots,n\}$ *such that* $\mathcal {A}_{i}[\alpha]$ *is weakly irreducible with* $\sigma(\mathcal {A}_{i}[\alpha])=\sigma(\mathcal {A}_{i})$ *for all* $i=1,2,\ldots,k$ *and*

4.2 $$ \big|\mathcal {A}_{i}[\alpha]\big|=\gamma_{i} \big|\mathcal {A}_{1}[\alpha]\big| D_{i}^{-(m-1)}\cdot \underbrace{D_{i}\cdots D_{i}}_{m-1},\quad i=2, \ldots,k, $$

*where* $\gamma_{i}>0$ *and* $D_{i}\in {\mathbb {R}}^{n \times n}$ *is a positive diagonal matrix*.

### Proof

As regards sufficiency, using Lemma 3.1 and Theorem 3.7, we have

$$\begin{aligned} \sigma\bigl(\mathcal {A}_{1}^{[r_{1}]}\circ \mathcal {A}_{2}^{[r_{2}]} \circ\cdots\circ \mathcal {A}_{k}^{[r_{k}]}\bigr)&\leq \sigma \bigl(\bigl( \mathcal {A}_{1}^{[r_{1}]}\circ \mathcal {A}_{2}^{[r_{2}]}\circ\cdots \circ \mathcal {A}_{k}^{[r_{k}]}\bigr)[\alpha]\bigr) \\ &=\sigma\bigl(\big| \mathcal {A}_{1}[\alpha]\big|^{[r_{1}]}\circ\big|\mathcal {A}_{2}[\alpha ]\big|^{[r_{2}]}\circ\cdots\circ\big|\mathcal {A}_{k}[\alpha]\big|^{[r_{k}]} \bigr) \\ &= \gamma_{2}^{r_{2}}\cdots\gamma_{k}^{r_{k}} \sigma\bigl(\big|\mathcal {A}_{1}[\alpha]\big|\bigr) \\ &= \sigma\bigl(\mathcal {A}_{1}[\alpha]\bigr)^{r_{1}}\sigma\bigl(\mathcal {A}_{2}[\alpha]\bigr)^{r_{2}}\cdots\sigma\bigl(\mathcal {A}_{k}[ \alpha]\bigr)^{r_{k}} \\ &=\sigma(\mathcal {A}_{1})^{r_{1}}\sigma(\mathcal {A}_{2})^{r_{2}} \cdots\sigma(\mathcal {A}_{k})^{r_{k}} \\ &\leq \sigma\bigl(\mathcal {A}_{1}^{[r_{1}]}\circ \mathcal {A}_{2}^{[r_{2}]} \circ\cdots\circ \mathcal {A}_{k}^{[r_{k}]}\bigr) \end{aligned}$$

and thus the sufficiency is true.

Necessarily, by (P1), without loss of generality, assume that $\mathcal {A}_{i}$ is nonnegative for all $i=1,2,\ldots,k$. Note that $\mathcal {C}=\mathcal {A}_{1}^{[r_{1}]}\circ \mathcal {A}_{2}^{[r_{2}]}\circ\cdots\circ \mathcal {A}_{k}^{[r_{k}]}$ is a strong $\mathcal {H}$-tensor by Theorem 2.3. Thus by Lemma 3.2, there exists $\alpha\subseteq\{1,2,\ldots,n\}$ such that $\mathcal {C}[\alpha]$ is a weakly irreducible strong $\mathcal {H}$-tensor with $\sigma(\mathcal {C})=\sigma(\mathcal {C}[\alpha])$. Consider that $\mathcal {C}[\alpha ]=(\mathcal {A}_{1}[\alpha])^{[r_{1}]}\circ\cdots\circ(\mathcal {A}_{k}[\alpha ])^{[r_{k}]}$. Thus $\mathcal {A}_{i}[\alpha]$ is a weakly irreducible strong $\mathcal {H}$-tensor for $i=1,2,\ldots,k$. Denote $\mathcal {B}^{[1-r_{k}]}=(\mathcal {A}_{1}[\alpha])^{[r_{1}]}\circ\cdots\circ(\mathcal {A}_{k-1}[\alpha ])^{[r_{k-1}]}$, which is weakly irreducible. Then $\mathcal {B}=(\mathcal {A}_{1}[\alpha])^{[\frac{r_{1}}{1-r_{k}}]}\circ\cdots\circ (\mathcal {A}_{k-1}[\alpha])^{[\frac{r_{k-1}}{1-r_{k}}]}$ is a weakly irreducible strong $\mathcal {H}$-tensor. Hence, by Theorem 3.7 and Lemma 3.1, we have

4.3 $$\begin{aligned}[b] \sigma(\mathcal {C})&=\sigma\bigl(\mathcal {B}^{[1-r_{k}]}\circ\bigl(\mathcal {A}_{k}[ \alpha]\bigr)^{[r_{k}]}\bigr)\geq\sigma(\mathcal {B})^{1-r_{k}} \sigma\bigl(\mathcal {A}_{k}[\alpha]\bigr)^{r_{k}} \\ &\geq \bigl(\sigma\bigl( \mathcal {A}_{1}[\alpha]\bigr)^{\frac {r_{1}}{1-r_{k}}} \cdots\sigma\bigl( \mathcal {A}_{k-1}[\alpha]\bigr)^{\frac {r_{k-1}}{1-r_{k}}}\bigr)^{1-r_{k}}\sigma\bigl( \mathcal {A}_{k}[\alpha]\bigr)^{r_{k}} \\ &=\sigma\bigl( \mathcal {A}_{1}[\alpha]\bigr)^{r_{1}}\cdots\sigma\bigl( \mathcal {A}_{k-1}[\alpha ]\bigr)^{r_{k-1}} \sigma\bigl(\mathcal {A}_{k}[ \alpha]\bigr)^{r_{k}} \\ &\geq\sigma(\mathcal {A}_{1})^{r_{1}} \cdots\sigma(\mathcal {A}_{k-1})^{r_{k-1}} \sigma(\mathcal {A}_{k})^{r_{k}}= \sigma(\mathcal {C}). \end{aligned} $$

Thus $\sigma(\mathcal {A}_{i}[\alpha])=\sigma(\mathcal {A}_{i})$ for all $i=1,2,\ldots,k$. Thus according to the observation that

$$\sigma\bigl(\bigl(\mathcal {A}_{1}[\alpha]\bigr)^{[r_{1}]}\circ\cdots \circ\bigl(\mathcal {A}_{k}[\alpha]\bigr)^{[r_{k}]}\bigr)=\sigma\bigl( \mathcal {A}_{1}[\alpha]\bigr)^{r_{1}}\cdots \sigma\bigl( \mathcal {A}_{k-1}[\alpha]\bigr)^{r_{k-1}} \sigma\bigl(\mathcal {A}_{k}[ \alpha]\bigr)^{r_{k}}, $$

where each $\mathcal {A}_{i}[\alpha]$ is a weakly irreducible strong $\mathcal {H}$-tensor, we use the induction on *k* to prove that (4.2) is true. Clearly, (4.2) is true for $k=2$ by Lemma 4.3. Assume that (4.2) is true for $k-1$. Now by (4.3) we have the following statements:

- $\sigma(\mathcal {B}^{[1-r_{k}]}\circ(\mathcal {A}_{k}[\alpha])^{[r_{k}]})= \sigma(\mathcal {B})^{(1-r_{k})} \sigma(\mathcal {A}_{k}[\alpha])^{r_{k}}$ and so, by Lemma 4.3, there exist $\gamma'_{k}>0$ and a positive diagonal matrix $D'_{k}\in {\mathbb {R}}^{n \times n}$ such that
  4.4 $$ \big|\mathcal {A}_{k}[\alpha]\big|=\gamma'_{k} | \mathcal {B}| \bigl(D'_{k}\bigr)^{-(m-1)}\cdot \underbrace{D'_{k}\cdots D'_{k}}_{m-1}. $$
- $\sigma(\mathcal {B})=\sigma(\mathcal {A}_{1}[\alpha])^{\frac {r_{1}}{1-r_{k}}} \cdots\sigma(\mathcal {A}_{k-1}[\alpha])^{\frac {r_{k-1}}{1-r_{k}}}$ and thus, by the induction assumption, we find that, for all $i=2,\ldots,k-1$, there exist $\gamma_{i}>0$ and a positive diagonal matrix $D_{i}\in {\mathbb {R}}^{n \times n}$ such that
  4.5 $$ \big|\mathcal {A}_{i}[\alpha]\big|=\gamma_{i} \big|\mathcal {A}_{1}[\alpha]\big| D_{i}^{-(m-1)}\cdot \underbrace{D_{i}\cdots D_{i}}_{m-1}. $$
- Using (4.4) and (4.5), we derive that there exist $\gamma_{k}>0$ and a positive diagonal matrix $D_{k}\in {\mathbb {R}}^{n \times n}$ such that
  $$\big|\mathcal {A}_{k}[\alpha]\big|=\gamma_{k} \big|\mathcal {A}_{1}[\alpha]\big| D_{k}^{-(m-1)}\cdot \underbrace{D_{k}\cdots D_{k}}_{m-1}. $$

Thus the result is proved. □

Next we characterize strong $\mathcal {H}$-tensors such that the equality of (3.6) holds in the case that $\sum ^{k}_{i=1}r_{i}>1$.

### Lemma 4.5

*Let* $\mathcal {A}\in \mathcal {R}^{(m,n)}$ *be a weakly irreducible strong* $\mathcal {H}$-*tensor and let* $t>1$. *Then* $\sigma(\mathcal {A}^{[t]})=\sigma(\mathcal {A})^{t}$ *if and only if* $n=1$.

### Proof

The sufficiency is trivial. Necessarily, $\mathcal {A}^{[t]}$ is obviously a weakly irreducible strong $\mathcal {H}$-tensor and thus, according to the proof of Lemma 3.6, (3.5) is true, *i.e.*,

$$\mathcal {M}\bigl(\mathcal {A}^{[t]}\bigr)z^{m-1}\geq\sigma(\mathcal {A})^{t}z^{[m-1]}= \sigma\bigl(\mathcal {A}^{[t]}\bigr)z^{[m-1]},\quad0< z= \bigl(x^{t}_{i}\bigr)\in {\mathbb {R}}^{n}, $$

from which it follows by Lemma 4.2 that

$$\mathcal {M}\bigl(\mathcal {A}^{[t]}\bigr)z^{m-1}=\sigma(\mathcal {A})^{t}z^{[m-1]}. $$

This means that the two Minkowski inequalities of (3.5) are equalities, and so, for all $i=1,\ldots,n$, there is at most one nonzero element for the elements

$$|a_{ii_{2}\ldots i_{m}}|x_{i_{2}}\ldots x_{i_{m}}, \quad\forall (i_{2},\ldots,i_{m})\neq(i,\ldots,i), $$

and there is at most one nonzero element for the two elements

$$\sum _{(i_{2},\ldots,i_{m})\neq(i,\ldots,i)}|a_{ii_{2}\ldots i_{m}}|x_{i_{2}}\ldots x_{i_{m}},\qquad|a_{ii\ldots i}|x_{i}^{m-1}-\sum _{(i_{2},\ldots,i_{m})\neq(i,\ldots,i)}|a_{ii_{2}\ldots i_{m}}|x_{i_{2}}\ldots x_{i_{m}}. $$

So, because of (3.4), we have, for all $i=1,\ldots,n$,

$$a_{ii_{2}\ldots i_{m}}=0, \quad\forall (i_{2},\ldots,i_{m})\neq (i,\ldots,i), $$

by considering the fact that $x_{i_{2}}\ldots x_{i_{m}}>0$, which means that $\mathcal {A}$ is diagonal. Recall that $\mathcal {A}$ is weakly irreducible. So, $n=1$. The result is proved. □

### Theorem 4.6

*Let* $\mathcal {A}_{1},\mathcal {A}_{2},\ldots, \mathcal {A}_{k}\in \mathcal {R}^{(m,n)}$ *be strong* $\mathcal {H}$-*tensors and let* $r_{1},r_{2},\ldots,r_{k}$ *be positive numbers such that* $\sum ^{k}_{i=1}r_{i}>1$. *Then*

$$\sigma\bigl(\mathcal {A}_{1}^{[r_{1}]}\circ \mathcal {A}_{2}^{[r_{2}]} \circ\cdots\circ \mathcal {A}_{k}^{[r_{k}]}\bigr)=\sigma( \mathcal {A}_{1})^{r_{1}}\sigma(\mathcal {A}_{2})^{r_{2}}\ldots \sigma(\mathcal {A}_{k})^{r_{k}} $$

*if and only if there exists* $\alpha\subseteq\{1,2,\ldots,n\}$ *with* $|\alpha|=1$ *such that* $\sigma(\mathcal {A}_{i}[\alpha])=\sigma(\mathcal {A}_{i})$ *for all* $i=1,2,\ldots,k$.

### Proof

As regards sufficiency, by considering $|\alpha|=1$, using Lemma 3.1 and Theorem 3.7, we have

$$\begin{aligned} \sigma\bigl(\mathcal {A}_{1}^{[r_{1}]}\circ \mathcal {A}_{2}^{[r_{2}]} \circ\cdots\circ \mathcal {A}_{k}^{[r_{k}]}\bigr)&\leq \sigma \bigl(\bigl( \mathcal {A}_{1}^{[r_{1}]}\circ \mathcal {A}_{2}^{[r_{2}]}\circ\cdots \circ \mathcal {A}_{k}^{[r_{k}]}\bigr)[\alpha]\bigr) \\ &= \sigma\bigl( \mathcal {A}_{1}[\alpha]\bigr)^{r_{1}}\sigma\bigl(\mathcal {A}_{2}[ \alpha ]\bigr)^{r_{2}}\cdots\sigma\bigl(\mathcal {A}_{k}[\alpha] \bigr)^{r_{k}} \\ &= \sigma(\mathcal {A}_{1})^{r_{1}}\sigma( \mathcal {A}_{2})^{r_{2}}\cdots\sigma (\mathcal {A}_{k})^{r_{k}} \\ &\leq\sigma\bigl(\mathcal {A}_{1}^{[r_{1}]}\circ \mathcal {A}_{2}^{[r_{2}]} \circ\cdots \circ \mathcal {A}_{k}^{[r_{k}]}\bigr), \end{aligned}$$

and thus the sufficiency is true.

Without loss of generality, assume that $\mathcal {A}_{i}$ is nonnegative for all $i=1,2,\ldots,k$. Note that $\mathcal {C}=\mathcal {A}_{1}^{[r_{1}]}\circ \mathcal {A}_{2}^{[r_{2}]}\circ\cdots\circ \mathcal {A}_{k}^{[r_{k}]}$ is a strong $\mathcal {H}$-tensor by Theorem 2.3. Thus, by Lemma 3.2, there exists $\alpha\subseteq\{1,2,\ldots,n\}$ such that $\mathcal {C}[\alpha]$ is a weakly irreducible strong $\mathcal {H}$-tensor with $\sigma(\mathcal {C})=\sigma (\mathcal {C}[\alpha])$. Set $t=\sum ^{k}_{i=1}r_{i}$ and $l_{i}=r_{i}t^{-1}$ for $i=1,2,\ldots,k$. Denote $\mathcal {B}=\mathcal {A}_{1}^{[l_{1}]}\circ \mathcal {A}_{2}^{[l_{2}]}\circ\cdots\circ \mathcal {A}_{k}^{[l_{k}]}$. Then $\mathcal {B}[\alpha ]$ is a weakly irreducible strong $\mathcal {H}$-tensor. Hence, by using Lemma 3.6, Theorem 3.7 and Lemma 3.1,

$$\begin{aligned} \sigma(\mathcal {C})&=\sigma\bigl(\mathcal {C}[\alpha]\bigr) =\sigma\bigl(\bigl(\mathcal {B}[\alpha] \bigr)^{[t]}\bigr) \geq\sigma\bigl(\mathcal {B}[\alpha]\bigr)^{t} \\ & \geq \bigl(\sigma\bigl(\mathcal {A}_{1}[\alpha]\bigr)^{l_{1}}\sigma \bigl(\mathcal {A}_{2}[\alpha ]\bigr)^{l_{2}}\cdots\sigma\bigl( \mathcal {A}_{k}[\alpha]\bigr)^{l_{k}}\bigr)^{t} \\ &=\sigma\bigl(\mathcal {A}_{1}[\alpha]\bigr)^{r_{1}}\sigma\bigl( \mathcal {A}_{2}[\alpha ]\bigr)^{r_{2}}\cdots\sigma\bigl( \mathcal {A}_{k}[\alpha]\bigr)^{r_{k}} \\ &\geq\sigma(\mathcal {A}_{1})^{r_{1}}\sigma(\mathcal {A}_{2})^{r_{2}} \cdots\sigma (\mathcal {A}_{k})^{r_{k}}=\sigma(\mathcal {C}), \end{aligned}$$

from which it follows that $\sigma(\mathcal {A}_{i}[\alpha])=\sigma(\mathcal {A}_{i})$ for all $i=1,2,\ldots,k$ and $\sigma((\mathcal {B}[\alpha])^{[t]})= \sigma(\mathcal {B}[\alpha])^{t}$, which implies by Lemma 4.5 that $|\alpha|=1$. The result is proved. □

## Conclusions

In this paper, we investigate the closure property of $\mathcal {H}$-tensors under the Hadamard product. It is shown that the Hadamard products of Hadamard powers of strong $\mathcal {H}$-tensors are still strong $\mathcal {H}$-tensors. We then bound the minimal real eigenvalues of the comparison tensors of the Hadamard products involving strong $\mathcal {H}$-tensors. Finally, we show how to attain the bounds by characterizing these $\mathcal {H}$-tensors.
